# Physicochemical properties and heavy metals characteristics of building ceramsites with oil-based drilling cutting residues

**DOI:** 10.1038/s41598-025-93394-7

**Published:** 2025-03-12

**Authors:** Deming Xiong, Chaoqiang Wang

**Affiliations:** 1 School of Physics, Chongqing University, Chongqing, 401331 China; 2 School of Electronics and Internet of Things, Chongqing Polytechnic University of Electronic Technology, Chongqing, 401331 China; 3 Chongqing Shanwaishan Blood Purification Technology Co., Ltd, Chongqing, 401123 China; 4 School of Material Science and Engineering, Chongqing Jiaotong University, Chongqing, 400074 China

**Keywords:** Oil based drilling cutting, Ceramsite, Physicochemical characteristic, Heavy metal, Solid Earth sciences, Pollution remediation

## Abstract

Oil-based drilling cutting residues (OBDCRs) are among the primary solid wastes generated during shale gas exploration and development. Utilizing existing equipment to transform OBDCRs into ceramsites appears to be a feasible and resource-efficient approach. In this study, building ceramsites were prepared with OBDCRs incorporating with fly ash (a byproduct of coal combustion) as raw materials. The aim was to comprehensively and systematically investigate physicochemical properties and characteristics of heavy metals (HMs) in the ceramsites. Research shows that building ceramsites can indeed be prepared using OBDCRs, which exhibit good comprehensive properties and strong resistance to acid/ alkali. The main HMs found in ceramsite are barium (Ba), chromium (Cr), nickel (Ni), lead (Pb), arsenic (As), cadmium (Cd), and mercury (Hg). During the calcination process, these OBDCRs, along with fly ash and foaming agent, underwent mutual melting, resulting in the formation of glass, anorthite and mullite. These newly formed phases effectively encapsulated HMs, resulting in varying degrees of enrichment of HMs such as As, Ba, Pb, Cr, and Ni, except for Cd and Hg. However, the leaching toxicity of these HMs in the ceramsite was significantly lower compared to that of the original OBDCRs. Further analysis revealed a significant increase in the proportion of Fe–Mn Oxides and Organic Matter in HMs such as Cr, Ni, As, Cd, and Pb, while the proportion of Exchangeable and Carbonates forms decreased markedly. This trend clearly demonstrated that the calcination process modified the physical and chemical properties of the ceramsite, and effectively stabilized HMs, i.e., migrated from an active state to a more stable form.

## Introduction

The shale oil and gas revolution has significantly altered the global energy landscape, extending the lifespan of the world’s petroleum industry and fostering a steady surge in global oil and gas reserves and production. This transformation has also profoundly influenced the energy strategies of numerous countries. In China, this revolution holds the promise of achieving the ambitious goal of “energy independence”^1^ . Nevertheless, the exploitation of shale oil and gas poses formidable environmental challenges.

During the drilling process, oil-based drilling fluids play a crucial role in preventing shale expansion, collapse, and blockage caused by hydration. While these fluids ensure the smooth operation of drilling, they also give rise to a significant issue: the generation of vast quantities of oil-based drilling cuttings (OBDCs). Currently, the primary method for disposing of OBDCs involves thermal desorption, resulting in the formation of oil-based drilling cutting residues (OBDCRs). Alarmingly, China alone produces up to 3 × 10^6^ tons of OBDCRs annually^2^. Previous studies have demonstrated that OBDCRs contain significant amounts of heavy metals (HMs) such as cadmium (Cd), chromium (Cr), mercury (Hg), lead (Pb), arsenic (As), nickel (Ni), and barium (Ba)^3–6^. These HMs are not only resistant to degradation but also highly toxic to biological systems. They can deteriorate soil structure and fertility^7,8^, disrupt the normal growth of plants and reproduction of microorganisms^7,9^, and ultimately upset the balance of the entire food chain^10^. Furthermore, HMs can diffuse into water bodies, contaminating rivers, lakes, and even groundwater, thereby posing a serious threat to aquatic organisms and human drinking water safety.

Currently, converting OBDCRs into ceramsite using existing equipment is a practical and resourceful method for addressing this issue, followed by the encapsulation and solidification of HMs. Ayati et al.^11^ conducted a study on the feasibility and performance of preparing lightweight aggregate from waste drill cuttings. Xie et al.^12^ explored the production of proppants using Cr-OBDCRs and found that they exhibited higher compressive strength and lower density compared to pure bauxite-based proppants. Xiong et al.^6^ investigated the physical properties, special contaminants, and environmental safety of porous ceramsite derived from OBDCRs. Wang et al.^13^ employed orthogonal experiments to study the influence of raw material composition, preheating temperature and time, as well as sintering temperature and time, on the performance of architectural ceramics. Li Yan et al.^14^ developed an oil-in-water emulsion separation ceramic membrane using OBDCRs and found it to exhibit excellent recyclability and corrosion resistance. Liu et al.^15^ investigated the preparation of high-strength and low-density proppants from OBDCRs and red mud. Yang et al.^16^ studied the influence of additives (V_2_O_5_ and MnO_2_) on the performance of the proppants and found that adding appropriate sintering additives could help improve the mechanical strength and acid resistance of ceramic proppant. However, most of studies have focused on using of OBDCRs as raw materials to produce ceramic products, and have overlooked the migration and transformation mechanism of HMs in ceramsite. Therefore, there is a need to clarify the content, speciation, and transformation mechanisms of HMs within the ceramsite to ensure its environmental safety and sustainable utilization.

This work aims to comprehensively assess the transformation mechanisms of HMs during the preparation of ceramsite from OBDCRs through field sampling and laboratory simulations. Advanced techniques such as scanning electron microscopy (SEM), X-ray diffraction (XRD), and energy dispersive spectroscopy (EDS), were employed to delve into the mineral composition, microstructure, and phase characteristics of ceramsite. Additionally, techniques like microwave digestion and inductively coupled plasma mass spectrometry (ICP-MS) were utilized to accurately quantify the content, speciation, and enrichment patterns of HMs in ceramsite. Through these investigations, the goal is to unravel the transformation mechanisms of HMs within OBDCRs during calcination, thereby providing a scientific rationale for the resourceful utilization of OBDCRs’ ceramsite.

## Materials and methods

### Sample collection and experimental methods

**Fig. 1:**
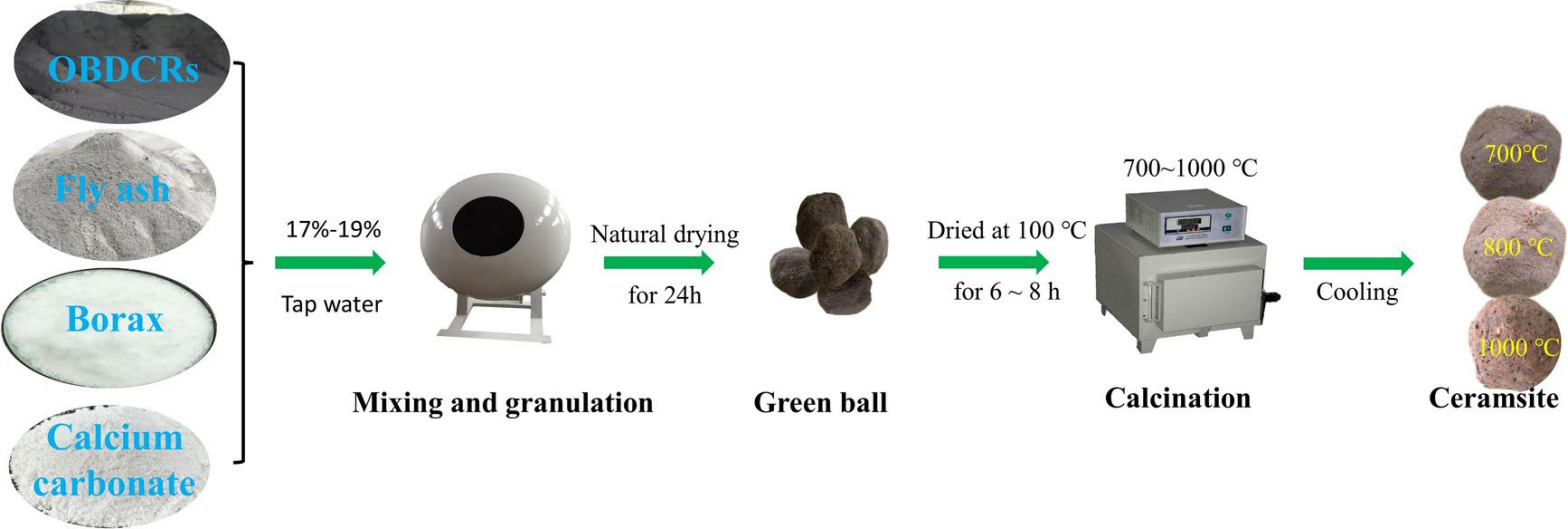
Process flow sheet of ceramsite preparation.

In this study, OBDCRs and fly ash served as the primary raw materials for the production of ceramsite. Borax functioned as a flux, while calcium carbonate acted as a foaming agent. The OBDCRs samples were sourced from various OBDCs recycling centers belonging to a shale gas company in Chongqing. Its chemical components are BaO(15.74%), SiO_2_(42.33%), SO_3_(10.40%), CaO(8.53%), Al_2_O_3_(10.56%), Fe_2_O_3_(4.00%), MgO(1.17%), Na_2_O(0.85%), K_2_O(2.39%), etc. The fly ash was kindly supplied by a power plant in Chongqing, and tap water was employed for both the mixing and curing processes. Its main chemical components are SiO_2_(43.3%), Al_2_O_3_(27.2%), CaO(7.48%), Fe_2_O_3_(10.2%), Na_2_O(2.22%), SO_3_(1.84%), MgO(2.53%), K_2_O(2.04%)etc. The flux utilized is borax, a white crystalline powder of analytical grade, boasting a purity level of 99.5%. Its chemical composition is represented by the formula Na_2_B_4_O_7_·10H_2_O. The foaming agent employed is calcium carbonate, another white crystalline powder of analytical grade, with a purity of 99.0%. The chemical formula for calcium carbonate is CaCO_3_. Both of these additives are readily available in the market and are manufactured by Tianjin Dengfeng Chemical Reagent Factory. These samples morphologies are shown in the following Fig. 1.

Figure 1 illustrates the elaborate preparation process of ceramsite. Initially, OBDCRs and fly ash undergo pretreatment procedures, including high-temperature calcination, crushing, grinding, and screening to a fine particle size of 200 mesh. Subsequently, the raw materials and auxiliary materials are accurately weighed and uniformly mixed (Table 1) to form spherical pellets ranging from 10 to 20 mm in diameter. These prototype balls are then naturally dried for a period of 24 h within a controlled indoor environment. Following drying, the prototype balls are placed inside a high-temperature box-type resistance furnace (muffle furnace) and sintered according to a predefined process system. After sintering, they are cooled to obtain the final ceramsite product. The heating temperatures of the resistance furnace were 700 ℃, 800 ℃, 850 ℃, 900 ℃, and 1000 ℃. The heating rate was 3 ℃/min, and the constant temperature time was 2 h. Finally, the calcination temperature decreased rapidly to 700 ℃, then decreased slowly to 300 ℃, and finally decreased rapidly to room temperature.

**Table 1:** Mix proportions of OBDCRs ceramsite (%, wt./wt.)

Specimen	Raw materials			
	OBDCRSs	Fly ash	Borax	Calcium carbonate
OPC-1	20	50	24	6.00
OPC-2	35	35	24	6.00
OPC-3	30	40	24	6.00
OPC-4	40	30	24	6.00
OPC-5	50	20	24	6.00

In this study, many trial experiments were conducted on the preparation of ceramsite using OBDCRs and fly ash. It can be seen that when the content of borax flux was greater than 30% and the calcination temperature reached above 1000 ℃, the ceramsite would undergo a melting phenomenon. When the borax content was less than 30%, the proportion of OBDCRs and fly ash could reach 70%, and the resulting ceramsite system belonged to the rich silicon-aluminum phase system. Therefore, the mix proportions of ceramsite were determined, and a total of 5 kinds of ceramsites were prepared (refer to Table 1). OBDCRs accounted for 20%, 30%, 35%, 40%, and 50% of the total weight of the samples, respectively.

### Test methods

#### X-ray fluorescence (XRF)

The elemental compositions of the ceramsite samples were precisely characterized by the PANalytical Axios Fast XRF spectrometer. This method employs primary X-ray photons or other microscopic ions to excite atoms within the material being tested. This excitation prompts the atoms to emit fluorescence, known as secondary X-rays, which are then utilized for the analysis of material composition and research on chemical states. The spectrometer was operated with an X-ray tube voltage of 60 kV and a current of 60 mA. The analytical crystals employed in the testing process included lif 200, ge111, pe 002, and PX1.

#### X-ray diffraction (XRD)

The material compositions of the raw materials, prototype samples, and ceramsite samples were determined through X-ray diffraction (XRD). This method relies on the diffraction patterns and intensities of a monochromatic X-ray beam in different directions within the crystal, enabling the identification of the mineral phases present in the raw materials, prototype samples, and ceramsite. The Cu-Kα radiation was operated at 35 kV and 60 mA, and the 2 θ range from 10° to 65° was scanned at a rate of 1.26°/min. The XRD spectrum was analyzed by the software of X’Pert HighScore and Plus MDI Jade 5.0.

#### Scanning electron microscopy (SEM)/energy dispersive spectrometer (EDS)

The SEM–EDS analytical testing combined the use of both SEM and EDS equipment to investigate the surface morphology and chemical composition of materials. The microstructure and morphology of the samples were analyzed by digital scanning electron microscope (SEM; ASTEREO SCAN440, Leica Cambridge, Ltd.). The Energy Dispersive Spectrometer (EDS) was employed to determine the types and concentrations of elements present in the samples.

#### Inductively coupled plasma mass spectrometry (ICP-MS)

The ICP-MS analytical instrument (Agilent 7800) was utilized to accurately measure the trace elements and HMs contents in the OBDCRs and ceramsite. The instrument was operated with an RF power of 1550 W, a nebulizer flow rate of 1 L/min, an auxiliary gas flow rate of 1 L/min, a pump rate of 20 r/min, and a sample flush time of 40 s. These parameters ensured precise and reliable measurements for the analysis of trace elements and HMs in the samples.

#### X-ray photoelectron spectroscopy (XPS)

XPS (Thermo Scientific K-Alpha) was used to analyze the valence states of HMs in samples. A suitable number of powdered samples was taken and placed on the sample tray, and then tested by XPS. The working conditions were a working voltage of 12 kV, a filament current of 6 mA, and a spot size of 400 μm. The pass energy of full spectrum was 150 eV, with a step size of 1 eV. For the narrow spectrum, the pass energy was 50 eV, with a step size of 0.1 eV. The energy standard using for binding energy correction is C_1_S (284.8 eV).

#### HMs’ content

The complete HMs content of OBDCRs, prototype balls, and ceramsite were tested and analyzed using the following method. Firstly, half a gram of the sample was placed in a 50 mL digestion vessel. Eight milliliters aqua regia and one milliliter hydrofluoric acid were added. The mixture was then placed on a graphite electric heating plate and digested at 150 °C for 20 to 60 min until complete digestion of the sample was achieved. After driving away the remaining acid in the digestion solution, the sample appeared as a light-yellow transparent droplet, slightly viscous. To prevent the formation of any unwanted impurities, the sample was cooled to room temperature with caution. Subsequently, the cooled sample was filtered and accurately diluted to a predetermined volume in a 50 mL volumetric flask. Once thoroughly mixed, the refined sample was subjected to analysis using the ICP-MS analytical instrument (Agilent 7800).

#### Leaching toxicity of HMs

The sulfuric acid-nitric acid method was employed to perform HMs leaching tests on OBDCRs, prototype samples, and ceramsites. This approach utilized a blend of concentrated sulfuric acid and concentrated nitric acid as an extractant, simulating the leaching of HMs in acidic rainfall environments. The objective was to assess the potential toxicity of these materials when exposed to acidic conditions, thereby providing valuable insights into their environmental impact. For details, please refer to the Chinese standard, *Sulfuric acid nitric acid method for leaching toxicity of solid waste*^17^ .

#### Physical properties tests

- (1) Bulk density
  1 $$\rho_{1} = \frac{{m_{1} \times 1000}}{V}$$
  where,* ρ*_*1*_ (kg/m^3^) is the bulk density; *m*_1_ (g) is the total weight of ceramsite; *V* (L) is the volume of ceramsite.
- (2) water absorption
  2 $$w = \frac{{m_{3} - m_{2} }}{{m_{3} }} \times 100\%$$
  where, *w* (%) is the water absorption; *m*_3_ (g) is the total weight of ceramsite after soaking in the distilled water for 1 h; *m*_2_ (g) is the total weight of ceramsite before soaking in the distilled water.
- (3) Cylinder compression strength test
  3 $$f_{n} = \frac{{p_{1} - p_{2} }}{A}$$
  where, *f*_n_ (MPa) is the compression strength of the cylinder; *P*_1_ (N) is the pressure value when the stamping die is pressed into 20 mm; *P*_2_ (N) is the mass of the stamping die; A (10,000 mm^2^) is the bearing area.
- (4) Porosity
  4 $$P = \frac{{\rho_{0} - \rho }}{{\rho_{0} }} \times 100\%$$
  5 $$\rho_{0} = \frac{{m_{3} }}{{m_{4} + m_{3} - m_{5} }} \times \rho_{w}$$
  where P(%) is the porosity of ceramsite,*ρ*_0_(g/cm^3^) is the density of ceramsite, *ρ*(g/cm^3^) is the volume density of ceramsite, *ρw*(g/cm^3^) is the water density. m_3_(g) is the initial mass of ceramsite, m_4_(g) is the mass of pyknometer containing ceramsite and liquid, m_5_(g) is the mass of the pyknometer filled with water.
- (5) Acid and alkali resistance
  6 $$R = \frac{{m_{a} }}{{m_{0} }}$$
  where R (%) is the acid and alkali resistance, m_0_ (g) is the mass of ceramsite before corrosion, and m_a_ (g) is the mass of ceramsite after corrosion.

#### HMs speciation

Samples such as prototype balls and ceramsite were naturally air dried in the laboratory. A planetary ball mill (QM-1sp) was used for grinding. The samples were stored in a plastic bottle. The experimental glass instruments were cleaned in an ultrasonic cleaner for a few minutes, and then rinsed three times with deionized water. Deionized water was prepared into the required reagent solution for the experiment. The experiment was conducted at room temperature. According to the Tessier^18^ analysis method, the specific method for testing HMs speciation was as follows:

1. Exchangeable(EXC). One-gram samples were extracted with 8 mL of 1 mol/L magnesium chloride solution (MgCl_2_, pH 7.0) for 1 h with continuous agitation at 25 ℃. The mixture was then centrifuged at 3000 rpm for 30 min, and the supernatant was filtered for further testing. The residue was subsequently cleaned with deionized water to remove residual solution.
2. Bound to Carbonates (CAR). The residue from (1) was leached with 8 mL of 1 mol/L sodium acetate solution (NaOAc, pH 5.0) at 25 ℃ for 5 h under continuous agitation. The centrifugation, filtration, and cleaning procedures were carried out in the same manner as in Step (1).
3. Bound to Fe–Mn Oxides (Fe–Mn). The residue from Step (2) was extracted with 20 mL of an NH_2_OH·HCl solution in 25% (v/v) acetic acid (HOAc). The extraction was performed at 96 ± 2 ℃ with occasional agitation for 6 h. Centrifugation, filtration, and cleaning operations were then conducted following the same protocol as in Step (1).
4. Bound to Organic Matter (ORG). The residue from Step (3) was treated with 3 mL of 0.02 mol/L HNO_3_ and 5 mL of 30% H_2_O_2_, adjusted to pH 2 with HNO_3_. The mixture was heated to 85 ± 2 ℃ for 2 h with intermittent agitation. An additional 3 mL of 30% H_2_O_2_ (pH 2 with HNO_3_) was added, and the sample was reheated to 85 ± 2 ℃ for 3 h with occasional agitation. After cooling, five milliliters of 3.2 mol/L NH_4_OAc in 20% (v/v) HNO_3_ were added, and the sample was diluted to 20 mL. Continuous agitation at 25 ℃ for 30 min followed. The centrifugation, filtration, and cleaning procedures were conducted as per the previous steps.
5. Residual (RES). The residue from Step (4) was digested with a mixed solution containing 4 mL of concentrated nitric acid and one milliliter of perchloric acid. The mixture was allowed to stand for 6 h, and the supernatant was collected for testing.

The liquid samples, which were filtered and extracted in each step, were stored at 2 ℃ and later analyzed using an ICP-MS analytical instrument (Agilent 7800).

#### HMs enrichment factors

To delve deeper into the characteristics of HM enrichment during the transformation of prototype balls into ceramsite, enrichment factors were incorporated into our study. The calculation formula for the HMs enrichment factor is as follows:

7 $$S=\frac{{\text{C}}_{1}-{\text{C}}_{0}}{{\text{C}}_{0}}$$

where S(%) is the enrichment factor of HMs, C_1_(mg/kg) is the content of HMs in ceramsite, and C_0_(mg/kg) is the content of HMs in prototype ball.

## Results and discussion

### Physical and chemical characteristics analysis

#### Morphology characteristics

**Fig. 2:**
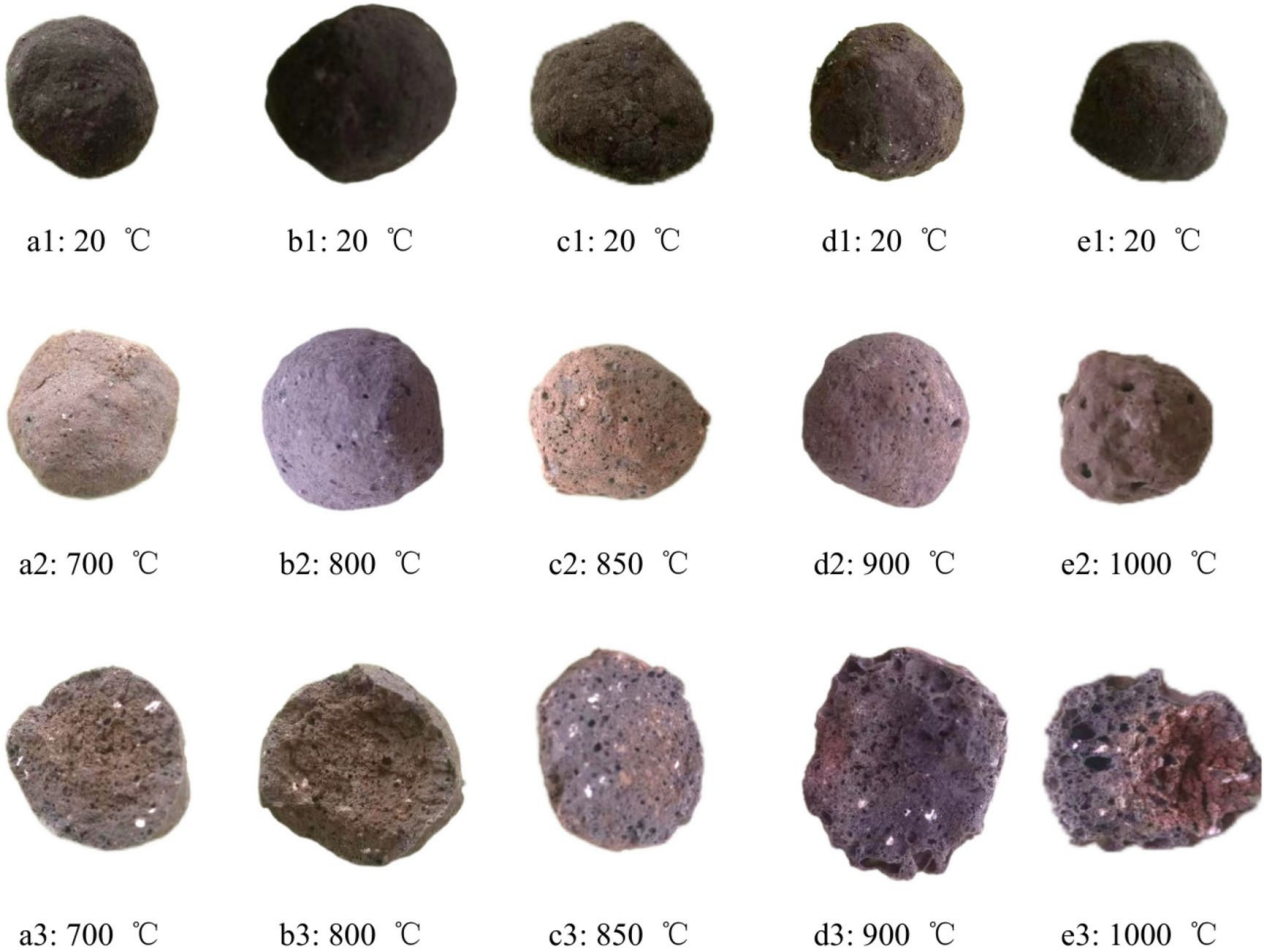
Morphological characteristics of ceramsites under different calcination temperatures.

Figure 2 illustrates the changes in appearance and morphology of ceramsites at different calcination temperatures. Initially, all prototype balls appeared as black-brown spheres with a diameter of approximately 2 cm (Fig. 2a1, Fig. 2b1, Fig. 2c1, Fig. 2d1, and Fig. 2e1). As the temperature increased, the surface color of the ceramsites darkened gradually, trending towards reddish-brown (Fig. 2a2, Fig. 2b2, Fig. 2c2, Fig. 2d2, and Fig. 2e2). Notably, Fig. 2a2 and Fig. 2b2 exhibited no visible bubbles on their surfaces. In contrast, Fig. 2c2 displayed more uniform small pores distributed across its surface. Figure 2d2 featured a smoother surface with a darker color and signs of melting, resulting in a slight reduction in ceramsite diameter. Figure 2e2 had melted, significantly decreasing in diameter, and showcasing many large pores with substantial spacing. Figure 2a3, Fig. 2b3, Fig. 2c3, and Fig. 2e3 depicted the internal pore distribution of the ceramsites. Figure 2a3 and 2b3 showed minimal large voids but some small pores, likely due to incomplete decomposition of the foaming agent at 800 ℃. At 850 ℃, Fig. 2c3 displayed numerous large, evenly distributed pores with uniform sizes. Further increasing the temperature to 900 ℃ resulted in some pores expanding significantly, leading to greater disparity in pore sizes. At 1000 ℃, the ceramsites exhibited a molten state, with pores showing a highly uneven distribution, especially at the edges where large pores were prominent.Throughout the process, it was evident that calcination at 850 ℃ allowed for effective decomposition of the foaming agent without causing melting, thereby preserving the integrity of the prototype balls and ensuring uniform pore distribution.

#### Chemical compositions of ceramsite

Table 2 clearly reveals that the main chemical components of prototype balls and ceramsite are the same, including SiO_2_, Al_2_O_3_, and CaO, which serve as the fundamental skeleton and filler components of the material. CaO is derived not only from OBDCRs but is also directly related to fly ash and calcium carbonate. BaO mainly comes from barite (BaSO_4_) in OBDCRs. This observation suggests that the chemical composition of the prototype balls remains relatively stable prior to and following the roasting process. However, it should be noted that the measured values exhibit fluctuations, which may be attributed to factors such as loss during the firing process and measurement inaccuracies^19–21^. These fluctuations are minimal and remain within a reasonable range^20^.

**Table 2:** Chemical composition of prototype ball and ceramsite (%, wt./wt.)

Samples	BaO	SiO_2_	SO_3_	CaO	Al_2_O_3_	Fe_2_O_3_	MgO	Na_2_O	K_2_O
Prototype ball	7.23	41.53	5.91	15.21	15.98	5.47	1.54	1.67	2.36
Ceramsite	9.23	37.56	7.49	12.35	14.07	5.67	1.32	7.34	2.26

#### Mineral composition analysis

**Fig. 3:**
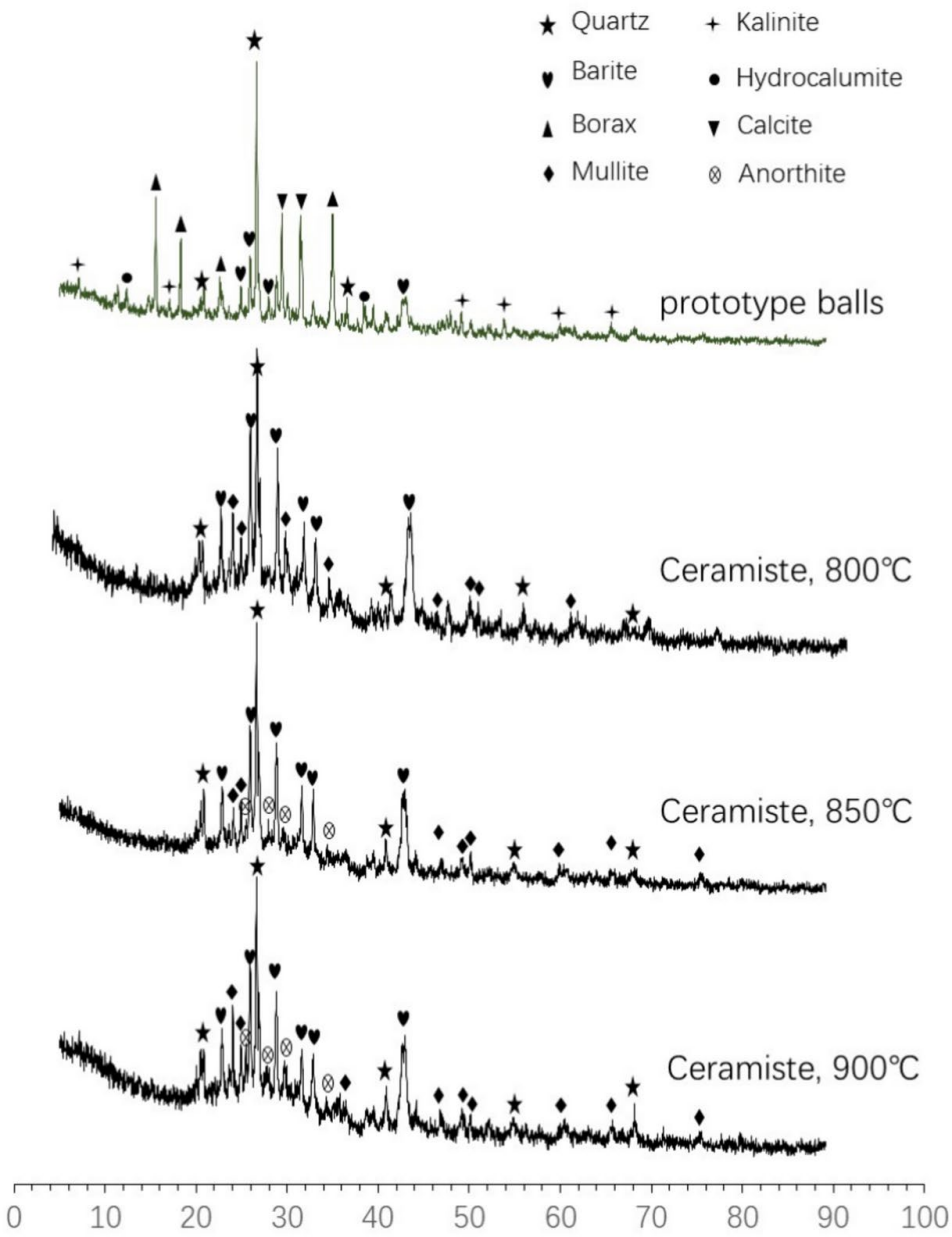
XRD patterns of prototype ball and ceramsite.

Figure 3 illustrates that the prototype ball primarily consists of quartz (SiO_2_), barite (BaSO_4_), borax (Na_2_B_4_O_7_·5H_2_O), calcite (CaCO_3_), aluminosilicate (Al_2_O_3_.2SiO_2_.2H_2_O), and kaolinite (Al_4_[Si_4_O_10_] (OH)_8_). The primary mineral components of ceramsite are quartz, barite, anorthite, and mullite. After undergoing high-temperature calcination, a significant transformation occurs in the mineral composition of ceramsite. Specifically, the quartz content decreases, while mineral peaks corresponding to borax and kaolin become weak and virtually undetectable. Conversely, the content of mineral phases such as anorthite, mullite, and glass increases. This shift is attributed to the melting of borax at high temperatures, which disrupts the internal silicate structure of quartz and kaolin, leading to partial melting and the formation of more complex aluminosilicate substances, such as anorthite, mullite, and glass. These changes not only enhance the strength of the ceramsite but also facilitate the encapsulation or immobilization of HMs within new phases.

#### Element analysis

**Fig. 4:**
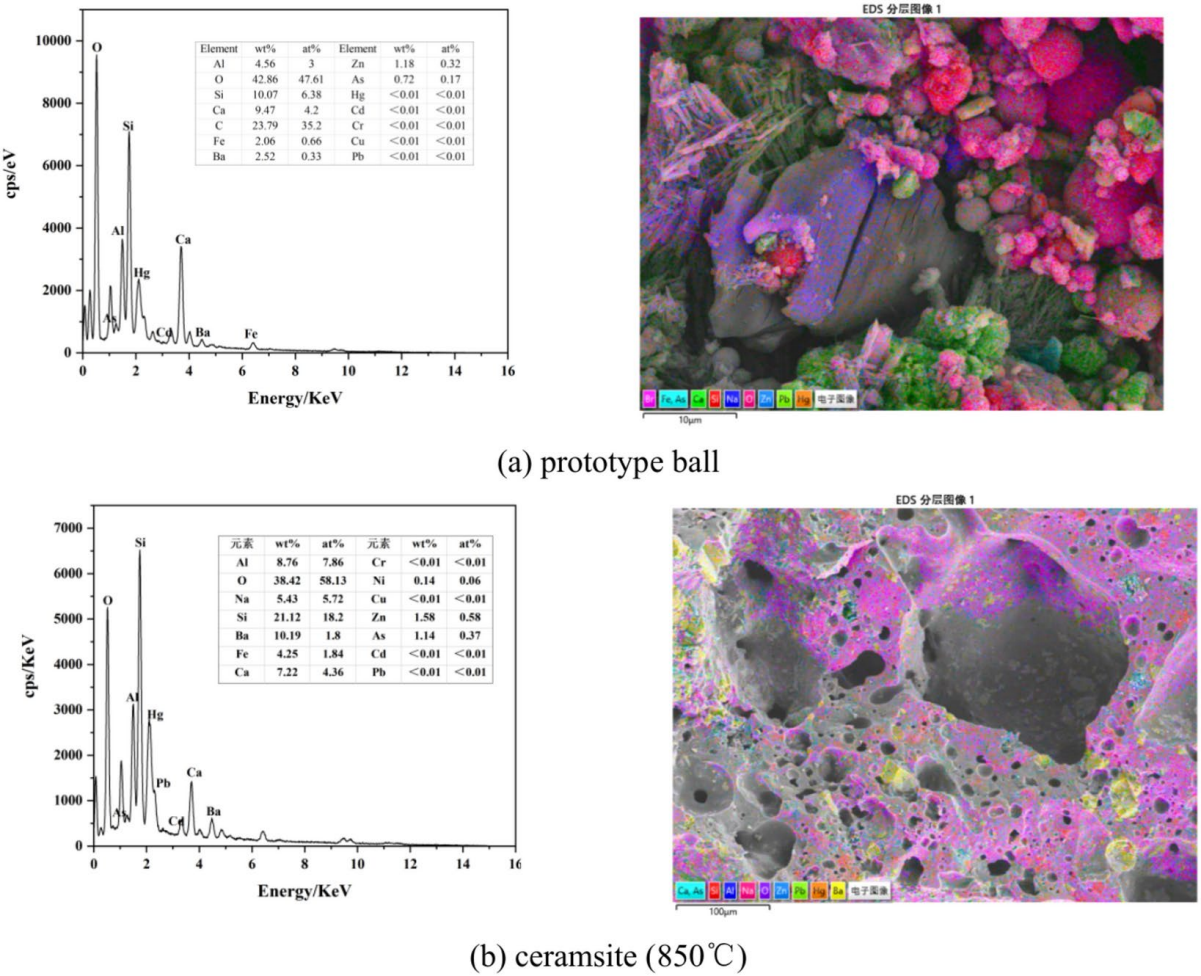
Microstructure and composition of prototype ball and ceramsite.

Figure 4a presents the microstructure and EDS spectrum of the testing area of the prototype ball, while Fig. 4b shows the corresponding microstructure and EDS spectrum of the ceramsite. Upon comparing Fig. 4a and b, it was evident that the primary elements of both the prototype ball and the ceramsite were Si, O, Ca, and Al. However, a notable difference was observed in the concentration of HMs. Specifically, the ceramsite exhibited a higher content of HMs such as Ba, Ni, Zn, and As compared to the prototype ball. This difference could be attributed to the high-temperature sintering process. During this process, a significant portion of Al and Si transformed into compounds like silicates or aluminosilicates. Additionally, the oxides of Ca, Na, and Fe, which were alkaline metal oxides, played a crucial role in facilitating the melting process. This not only enhanced the completeness of reactions among various components in the ceramsite but also promoted the formation of more liquid phases^22^.

Furthermore, the increase in the content of HMs like Ba, Ni, Zn, and As in the ceramsite could be explained by the presence of glass phases in the selected EDS testing area. These glass phases encapsulated the HMs within the ceramsite, effectively preventing their leaching and ensuring a safer and more environmentally friendly end product.

#### Microstructure analysis

**Fig. 5:**
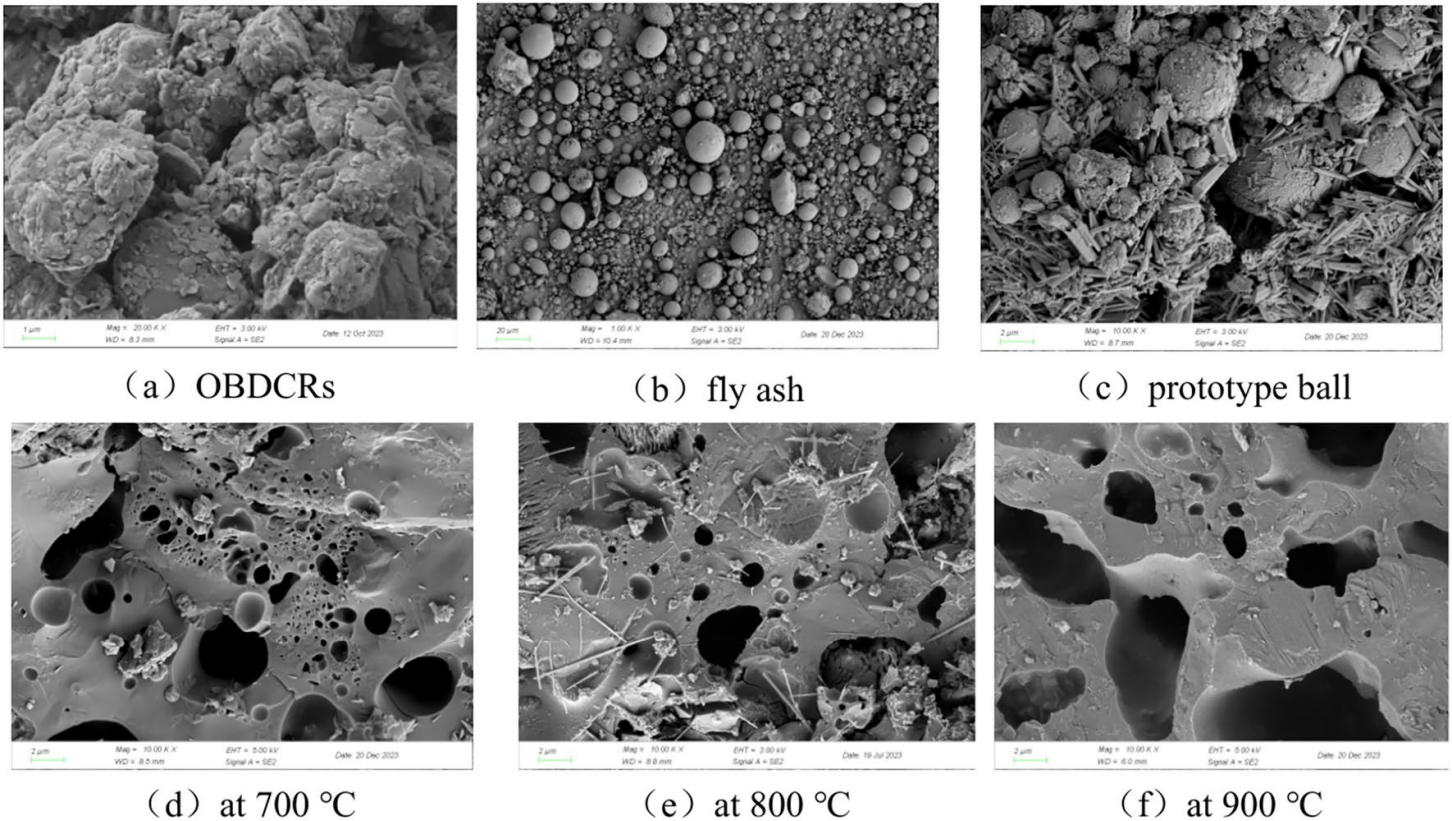
The microstructure of OBDCRs, fly ash, prototype ball and ceramsite.

After drying and grinding fly ash, OBDCRs, prototype balls, and ceramsite into powder, their internal microstructure was examined using scanning electron microscopy, yielding the test results presented in Fig. 4 and 5.

Figure 5a demonstrates the variability in size and irregular shape of OBDCRs. The surface of these particles exhibits a rough and uneven texture, with numerous protrusions and depressions. Additionally, a minor amount of layered CaO could be discerned within the OBDCRs, and numerous small particles adher to their surfaces. Figure 5b illustrates the distinct spherical morphology of fly ash, a characteristic trait typical of this material. Due to the variation in particle sizes, larger particles of fly ash are observed to have numerous smaller spherical particles adhered to their surfaces. Figure 5c reveals the microstructure of the prototype balls, showing a relatively loose internal structure with fly ash and OBDCRs embedded within each other. The spherical particles represent fly ash, while the polygonal-shaped samples correspond to OBDCRs. The columnar formations are calcium carbonate. Notably, numerous small powder-like particles are present on the surface of the material phase, attributed to the fact that the prototype balls had not undergone high-temperature calcination, resulting in the independent existence of various chemical components within the prototype body.

In Fig. 4a, spherical particles represent fly ash, and the irregularly shaped components are primarily composed of OBDCRs. Notably, regions with a higher concentration of fly ash appear brighter, followed by those containing OBDCRs. In contrast, Fig. 4b reveals a significant change in the microstructure of the sample, where independent particles such as fly ash and OBDCRs are no longer distinguishable. Instead, they have fused together, leading rise to the formation of numerous pores within the ceramsite.

Figure 5d, e, and f display the microstructure of ceramsite, characterized by solid phases and pores. With increasing temperature, a clear trend emerged: the number of pores in the ceramsite increased gradually, along with an enlargement in pore diameter. The larger pores in Fig. 5f can be attributed to various factors. Firstly, the evaporation of organic matter within the particles contributed to the formation of these pores. Secondly, the decomposition of carbonates released gases that formed additional pores. Lastly, the evaporation of water from the raw materials further enhanced the porosity. Conversely, smaller pores were formed during the sintering process when gas became trapped within the molten liquid phase, resulting in a smoother surface texture for these smaller pores.

#### Performance analysis

**Fig. 6:**
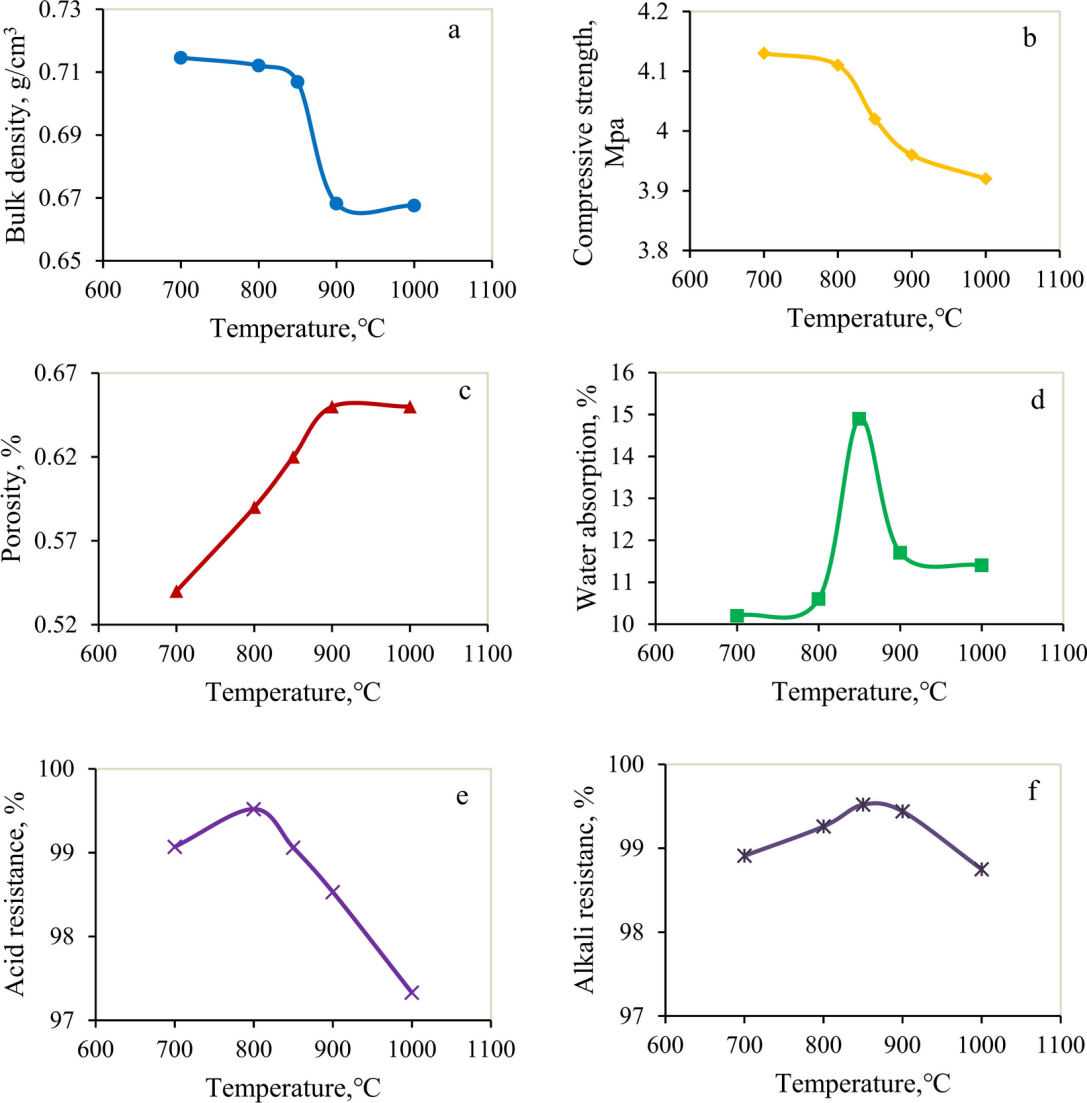
Performance of ceramsites under different calcination temperatures.

As depicted in Fig. 6, a clear trend emerged: as the temperature increased, the bulk density and compressive strength decreased while the porosity gradually increased. Simultaneously, the water absorption rate, acid resistance and alkali resistance showed a trend of initially increasing and then decreasing. These changes offer a clear understanding of the influence of calcination temperature on the properties of ceramsites.

##### (1) Bulk density and Compressive strength

Figure 6a illustrates that the bulk density decreased with increasing temperature. This was attributed to the enhanced decomposition of the foaming agent, which generated a higher volume of bubbles, expanding the material’s volume while reducing its mass per unit volume. Additionally, the proliferation of pores contributed to a less compact internal structure, thus weakening the ceramsite’s compressive strength (Fig. 6b). This decrease in strength stemmed from the reduced effective bearing area caused by the presence of pores, making the material more susceptible to deformation and failure under external forces.

##### (2) Porosity

The increase in porosity was caused by the rise in the number of bubbles in the ceramsite (Fig. 6c). At higher temperatures, the reaction rate of the foaming agent accelerated, leading to the generation of more bubbles. These bubbles created numerous pores, consequently enhancing the porosity. The enhancement of porosity was highly significant for the thermal insulation properties and lightweight nature of ceramsites.

##### (3) Water absorption

The water absorption rate of ceramsite followed a distinct variation pattern, initially increasing and then decreasing as the temperature rose (Fig. 6d). At lower temperatures, the ceramsite contained numerous minute pores that effectively facilitated water penetration into its interior. As the temperature gradually increased, these pores grew in both number and size, resulting in enhanced water infiltration channels and an expanded water storage capacity. Consequently, the water absorption rate increased. However, as the temperature continued to rise, some pores ruptured or coalesced due to excessive expansion. This phenomenon diminished the water infiltration channels and reduced the available storage space, ultimately leading to a decline in the water absorption rate. These observations provide a nuanced understanding of how calcination temperature influences the water absorption characteristics of ceramsite.

##### (4) Acid resistance and alkali resistance

It was noteworthy that ceramsite, when calcined under varying temperature conditions, consistently demonstrated robust acid and alkali resistance (Fig. 6e and f). This suggests that the calcination temperature had a relatively minor impact on their acid and alkali stability. Instead, the main factors influencing their resilience were the inherent chemical composition and microstructure of the ceramic material itself.

In summary, when the calcination temperature was 850 ℃, the bulk density of ceramsites was 707.10 kg/m^3^, the water absorption rate was 10.9%, the compression strength could reach 4.08 MPa, and the porosity was 0.6%, indicating that the pore size distribution was more uniform. This resulted in better comprehensive performance and improved acid and alkali resistance.

### Analysis of HM’s characteristics

#### HM’s content

As illustrated in Fig. 4, the prototype ball and ceramsite contained various elements, with HMs being the primary concern due to their potential environmental harm. Consequently, a comprehensive analysis of HMs content was conducted on OBDCRs, prototype balls, and ceramsite. The results, presented in the attachment (Appendix 1), reveal notable differences in the average concentration of HMs in the samples. Specifically, the table highlights the presence of barium (Ba), chromium (Cr), nickel (Ni), lead (Pb), arsenic (As), cadmium (Cd), and mercury (Hg), with Cd and Hg exhibiting the lowest concentrations. Notably, the content of HMs such as Ba, Ni, Pb, Cd, and Ba in OBDCRs surpassed the local soil background value, indicating a potential risk of HMs pollution. These findings underscore the importance of careful monitoring and management of these materials to minimize environmental impact.

#### HM’s leaching behavior

According to the standard^17^, leaching toxicity tests for HMs were conducted on OBDCRs, fly ash, prototype balls, and ceramsites. The results of these tests were summarized in the attachment (Appendix 2). It was evident that after high-temperature sintering, the leaching toxicity of HMs in ceramsite was significantly lower than that observed in OBDCRs^23^.

To further elucidate this phenomenon, we draw upon the findings from XRD and SEM analysis conducted both before and after calcination. These analyses indicated that the HMs present in OBDCRs were effectively encapsulated by the glass phase. This encapsulation occurred due to the destruction of the raw material’s internal structure during the sintering process, which led to partial melting. As a result, some HMs became encapsulated within the glass phase, thereby reducing their leaching concentration.

Glass played a pivotal role in the solidification of HMs within ceramsite. Its presence not only helped to encapsulate these harmful elements but also contributed to the overall stability and durability of the ceramsite.

#### HMs’ speciation

##### (1) HM’s speciation

According to Tissier’s^18^ theory, the bioavailability and ecological toxicity of HMs are directly influenced by their chemical speciation. These metals exist in five distinct speciations: EXC, CAR, Fe–Mn, ORG, and RES. EXC and CAR exhibit high mobility in water and soil, resulting in their high bioavailability, making them the most easily absorbed speciation of HMs by organisms. CAR is particularly prone to absorption in plants or water systems and is considered a directly effective components in terms of its environmental impact. In a reducing environment, such as that caused by acid rain, Fe–Mn can undergo a transformation, easily converting into EXC. This conversion allows these metals to enter water and soil, thus contaminating the environment^24^. ORG, on the other hand, is a relatively stable compound with low activity and solubility. This stability reduces its absorption by plants, and therefore, its environmental hazards are relatively low. RES is typically entrenched in the lattice of silicates. Under normal natural conditions, they are not easily released. This stability ensures that they can persist in sediments for extended periods, are not easily absorbed by plants, and are resistant to migration and transformation^25^.

##### (2) Content of HMs

The distribution of HMs in ceramsite displayed distinct trends. In the prototype balls, the total HMs’ content ranked from highest to lowest as follows: Ba, Cr, Ni, Pb, As, Cd, and Hg. Notably, Ba exhibited the highest concentration, reaching 2873.78 mg/kg, while Cr and Ni also displayed relatively high levels, with concentrations of 49.59 mg/kg and 43.76 mg/kg, respectively. After transforming into ceramsite, the overall HMs’ content maintained a similar sequence, with Ba remaining dominant and reaching a higher concentration of 3299.17 mg/kg. Cr and Ni continued to be present at significant levels, with concentrations of 43.32 mg/kg and 33.65 mg/kg, respectively. Interestingly, after high-temperature calcination, the total HMs’ content in the ceramsite increased for most elements, except for Cd and Hg. This finding indicated the enrichment of HMs within the ceramsite during the calcination process.

##### (3) Distribution characteristics of HMs’ speciation

**Fig. 7:**
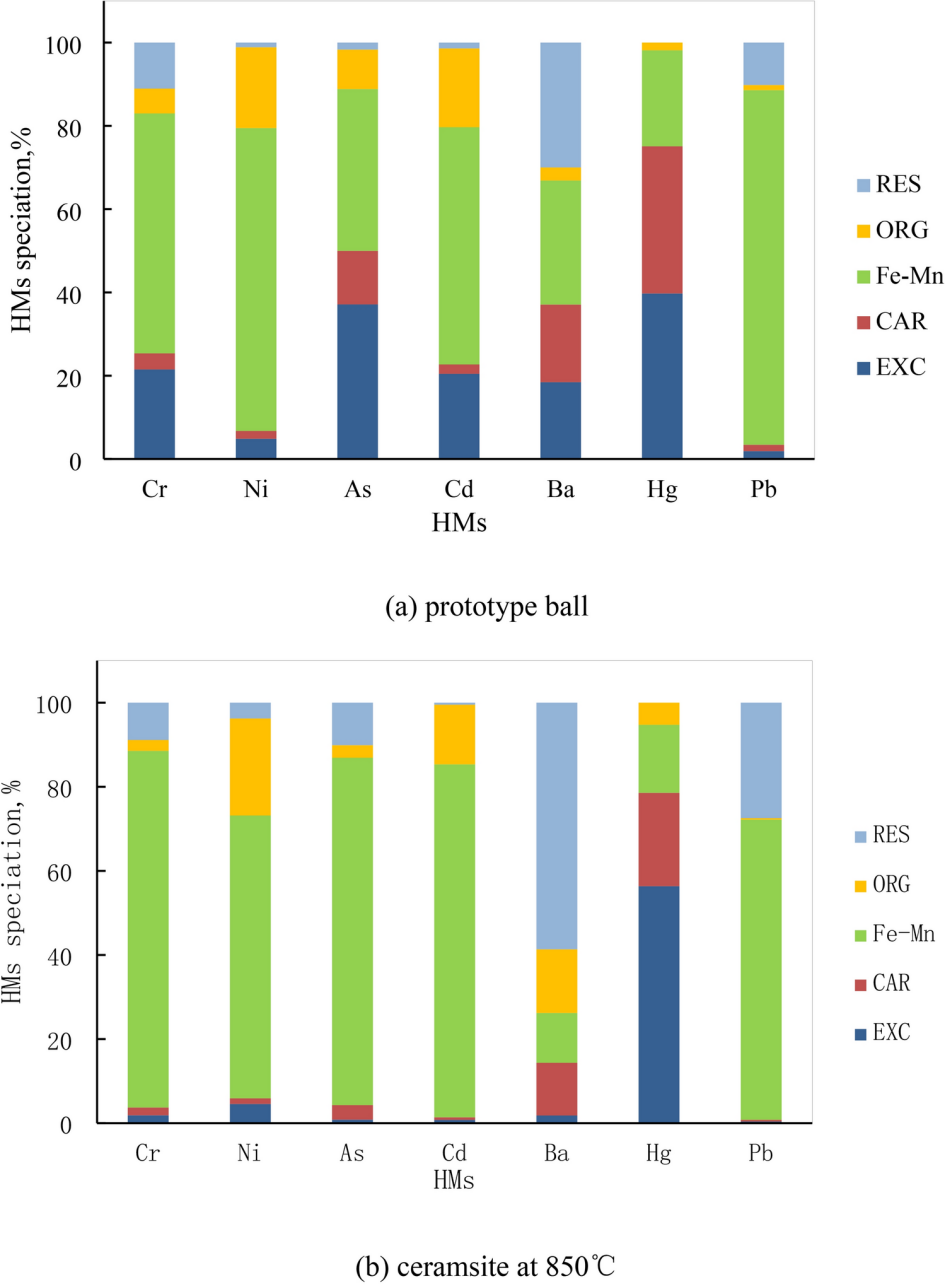
HMs’ speciation in prototype ball and ceramsite.

The speciation of HMs in both the prototype ball and ceramsite was clearly depicted in Fig. 7. For the prototype ball, the Fe–Mn compound content dominated the speciation of Cr, Ni, As, Cd, Ba, and Pb, accounting for 57.62%, 72.73%, 38.85%, 56.98%, 29.80%, and 85.17%, respectively. In contrast, Hg exhibited the highest EXC content, constituting 39.75%. When it came to ceramsite samples, the Fe–Mn content remained the most significant for Cr, Ni, As, Cd, and Pb, with percentages of 84.82%, 67.26%, 82.61%, 83.94%, and 71.38%, respectively. However, Ba stood out with the highest RES content, accounting for 58.64%. Similarly, Hg maintained its high EXC content, making up 56.36%. Based on these observations, it could be deduced that numerous thermochemical reactions, primarily high-temperature oxidation reactions, occurred during the production of ceramsite by OBDCRs. Consequently, in ceramsite, the proportion of Fe–Mn for Cr, Ni, As, Cd, and Pb significantly increased. Concurrently, the content of EXC and CAR decreased notably, while the content of ORG and RES rose. This shift indicated that HMs migrated from more active forms to more stable ones. The abrupt surge in the RES content of Ba particularly suggested that Ba was prone to transforming into a stable form. In summary, after undergoing high-temperature calcination, the majority of HMs within ceramsite transformed into stable forms, such as Fe–Mn, ORG, and RES, thereby enhancing the safety and stability of ceramsite products. The HMs in the ceramsite have been solidified and are not easily absorbed by plants, and have resistance to migration and transformation^21–23^.

#### HMs’ enrichment

The enrichment factor is a crucial tool for evaluating and analyzing the enrichment of HMs in prototype balls and ceramsite. The data presented in Table 3 revealed that ceramsite exhibited varying degrees of HMs enrichment, with As, Ba, Ni, Pb, and Cr ranking from the highest to the lowest enrichment levels. As and Ba belonged to medium enrichment levels, while Cr, Ni, and Pb belonged to minimal enrichment levels. The extent of enrichment in ceramsite was influenced by the physical attributes of HMs, specifically their melting and boiling points^26^.Cd and Hg had melting and boiling points lower than the calcination temperature. As a result, during the calcination process, a significant amount of Cd and Hg evaporated, leading to a notable difference in their total quantity before and after sintering. Specifically, Cd decreased by 53.36%, while Hg decreased by 23.10% (Table 3). Therefore, no enrichment of Cd and Hg was observed in the ceramsite.

**Table 3:** Migration and transformation of HMs in prototype balls and ceramsite.

Metals	C_0_, mg/kg	C_1_, mg/kg	S	Enrichment level
Cr	49.59	53.32	0.08	Minimal
Ni	43.76	47.65	0.09	Minimal
Pb	24.21	26.56	0.09	Minimal
As	21.14	26.82	0.27	Medium
Ba	2873.78	3299.17	0.15	Medium

*Enrichment level^22^: minimal (0 < s < 0.1), medium (0.1 < s < 0.8), maximal (s > 0.8).

#### Analysis of XPS

**Fig. 8:**
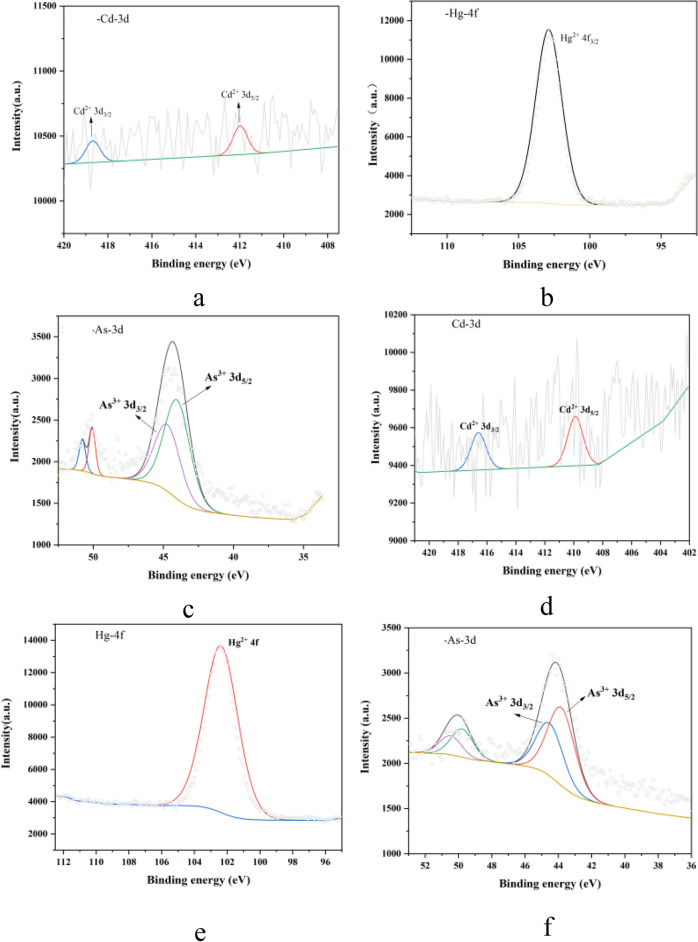
Valence states of HMs in prototype balls and ceramsite.

The valence states of Cd, Hg, and As in prototype balls and ceramsites were carefully analyzed through XPS testing to gain deeper insights into their toxicity. The Avantage software was used to fit the XPS spectra of these HMs. As depicted in Fig. 8a, b, and c, the XPS spectra of Cd, Hg, and As in the prototype balls revealed their valence states. Specifically, Cd was primarily present in its bivalent form, characterized by binding energies of 411.98 eV for 3d_3/2_ and 418.68 eV for 3d_5/2_. Hg existed in its divalent state with a binding energy of 102.88 eV, while As adopted a trivalent form, exhibiting binding energies of 44.02 eV for 3d_3/2_ and 44.72 eV for 3d_5/2_. These findings confirmed that in prototype balls, Cd, Hg, and As were present as Cd^2+^, Hg^2+^, and As^3+^, respectively. Figure 8d, e, and f illustrated the valence states of Cd, Hg, and As in ceramsites. Cd retained its divalent form, with binding energies of 409.89 eV for 3d_3/2_ and 416.59 eV for 3d_5/2_. Hg also maintained its divalent state, exhibiting a binding energy of 102.38 eV. Similarly, As persisted in its trivalent form, with binding energies of 43.83 eV for 3d_3/2_ and 44.52 eV for 3d_5/2_. This indicated that after high-temperature calcination, the valence states of these HMs in the prototype balls did not undergo significant changes, retaining their Cd^2+^, Hg^2+^, and As^3+^ forms in the ceramsites.

### Analysis of mechanism

#### Preparation mechanism of ceramsite

**Fig. 9:**
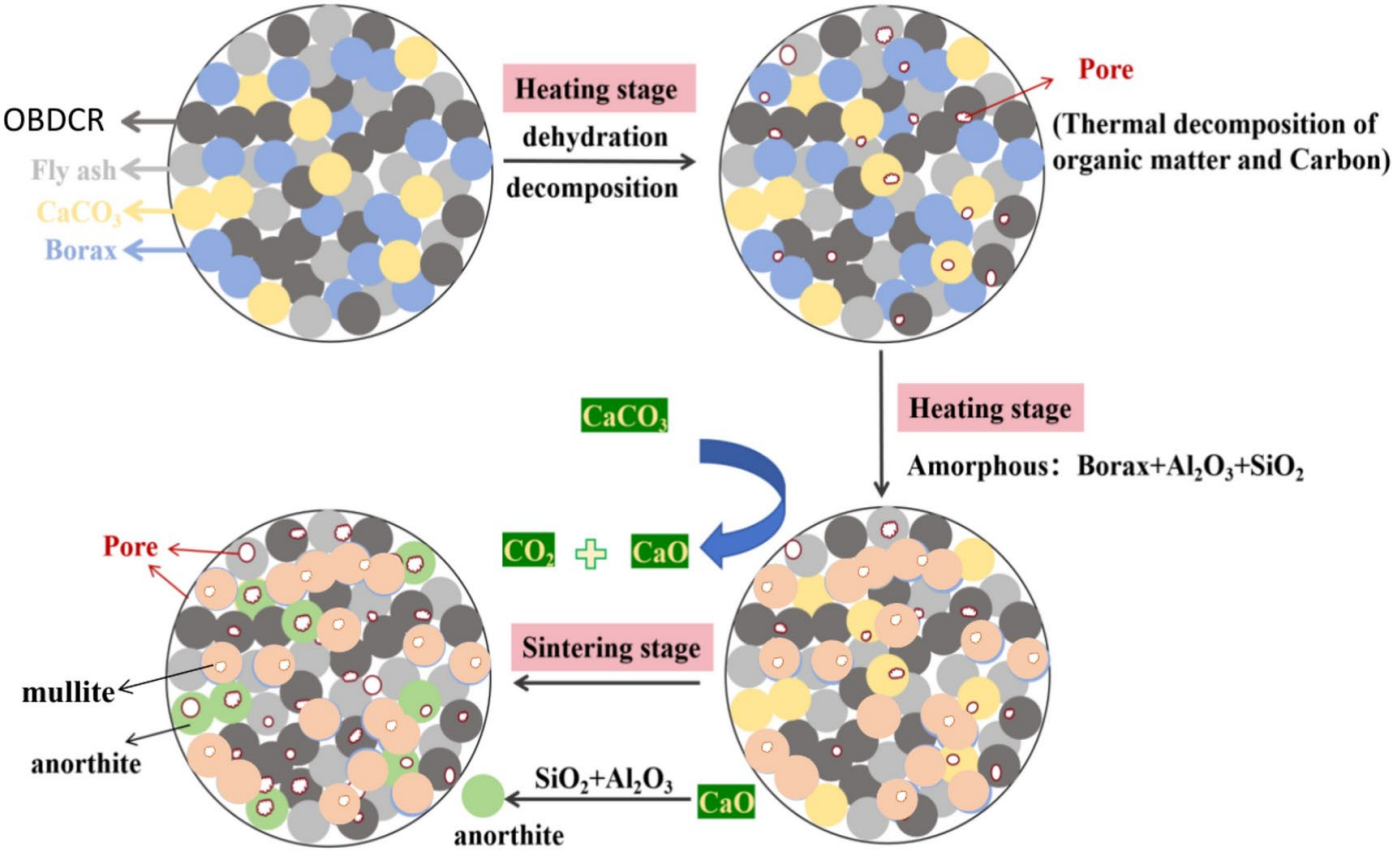
Component evolution, and HMs’ solidification mechanism in ceramsite.

In Fig. 9, the black ball signifies OBDCR, the gray ball represents fly ash, the yellow ball denotes calcium carbonate, the blue ball stands for borax, the green ball indicates anorthite, the orange ball represents mullite. Figure 9 illustrates the mechanism of preparing ceramsite from OBDCRs. In this process, a key component is the foaming agent (CaCO_3_), which plays a vital role in creating of pores within the ceramsite. The foaming agent decomposes within the temperature range of 650 ℃ to 1000 ℃, releasing CO_2_ and CaO. During the initial heating stage, the water present in the prototype ball gradually evaporates, some organic compounds undergo volatilization and decomposition reactions, and carbon undergoes oxidation reactions. The generated CO_2_ and water create a small number of pores in situ. As the temperature steadily increases, the flux (borax) initiates its decomposition, yielding Na_2_O and B_2_O_3_. These resulting components react with the majority of oxides, forming compounds with low melting points. These compounds effectively dismantled the silicate structures of minerals, including quartz and kaolin, ultimately leading to the gradual transition of the body into a molten state. Upon entering the constant temperature stage, the liquid phase generated through the melting process encapsulates the foaming agent, which decomposes completely, resulting in the formation of numerous pores. Simultaneously, SiO_2_ and Al_2_O_3_ react to form mullite. The CaO produced from the decomposition of CaCO_3_ reacts with the unmelted silicon aluminum oxides, creating a small quantity of anorthite.

#### Mechanism of HMs solidification

The solidification mechanism of HMs in ceramsite could be understood by the solidification of the glass phase and the solidification of the crystalline phase^27^. High-temperature calcination triggered the melting of the ceramsite’s skeleton components, where SiO_2_ and Al_2_O_3_ reacted with other chemical constituents. This melting and subsequent chemical reactions led to the formation of a more stable form of HMs. This stabilization process significantly improved the resistance of HMs against migration and leaching. This process also produced a significant amount of anorthite, mullite, and glass, which played a vital role in immobilizing HMs. The glass phase effectively encapsulated HMs into the amorphous glass network, sealing them within a ceramsite matrix to prevent their release into the environment. Anorthite had the capability to immobilize HMs within its lattice structure through isomorphic substitution. The solidification mechanism in the anorthite phase further reduced the leaching concentration of HMs^22^.

## Conclusion

In this paper, laboratory experiments were conducted to simulate the preparation of ceramsite through the calcination of OBDCRs and fly ash. The objective was to comprehensively and systematically investigate the physicochemical properties and characteristics of HMs in ceramsite.

1. Research has shown that the main chemical components of ceramsite are SiO_2_, Al_2_O_3_, and CaO. After high-temperature calcination, the surface of the ceramsite changes from black-brown to reddish-brown. The pore size distribution of ceramsite becomes more uniform, exhibiting better comprehensive performance and improved acid and alkali resistance at 850 ℃.
2. The main HMs in ceramsite were As, Ba, Ni, Pb, Cr, Cd, and Hg. During the calcination process, these OBDCRs, along with fly ash and a foaming agent, underwent mutual melting, resulting in the formation of glass, anorthite, and mullite. These newly formed phases effectively encapsulated the HMs, resulting in varying degrees of HMs enrichment in As, Ba, Pb, Cr, and Ni, except for Cd and Hg. However, the leaching toxicity of these HMs in the ceramsite was significantly lower compared to that of the original OBDCRs. This indicates that the calcination process not only modified the physical and chemical properties of the ceramsite but also effectively stabilized the HMs.
3. Further analysis revealed a marked increase in the proportion of Fe–Mn and RES fractions in HMs such as Cr, Ni, As, Cd, and Pb, while the EXC and CAR fractions decreased significantly. This trend clearly demonstrates that during high-temperature calcination, HMs transitioned from an active state to a more stable form, thereby reducing their potential environmental risks.

## Supplementary Information

Supplementary Information.

## Data Availability

All data generated or analysed during this study are included in this published article.
