# Supplementary material for: Physicochemical properties and heavy metals characteristics of building ceramsites with oil-based drilling cutting residues
Source: Sci Rep. 2025 Mar 12;15:8473. doi: 10.1038/s41598-025-93394-7 (PMC11897375; doi:10.1038/s41598-025-93394-7)
Supplement: Supplementary file 1 — Supplementary Information. [file 41598_2025_93394_MOESM1_ESM.docx]

Appendix 1

The average content of HMs in OBDCRs, prototype balls, ceramsite and soil

| Samples | Cr，mg/kg | Ni，mg/kg | Pb，mg/kg | Hg，mg/kg | Cd，mg/kg | As，mg/kg | Ba，mg/kg |
| --- | --- | --- | --- | --- | --- | --- | --- |
| OBDCRs | 32.23 | 59.10 | 36.93 | 0.001 | 3.83 | 13.80 | 3509.67 |
| Prototype balls | 49.59 | 43.76 | 24.21 | 0.19 | 1.03 | 21.14 | 2873.78 |
| Ceramsite | 43.32 | 33.65 | 26.56 | 0.09 | 0.76 | 26.82 | 3299.17 |
| Soil | 90.00 | 40.00 | 35.00 | 0.15 | 0.20 | 15.00 | 0 |

Appendix 2

Leaching behavior of HMs in OBDCRs, fly ash, prototype balls, and ceramsite

| HMs | OBDCRs, mg/ L | fly ash, mg/ L | Prototype balls, mg/ L | Ceramsite, mg/ L | National standard,mg/L |
| --- | --- | --- | --- | --- | --- |
| Cr | 0.019 | 0.609 | 0.348 | 0.279 | 1.50 |
| Ni | 1.529 | 0.429 | 0.626 | 0.209 | 1.00 |
| As | 0.096 | 0.047 | 2.667 | 0.040 | 0.50 |
| Cd | 0.235 | 0.013 | 0.040 | 0.006 | 0.10 |
| Hg | 0.004 | 0.004 | 0.001 | 0.001 | 0.05 |
| Pb | 0.094 | 0.017 | 0.070 | 0.032 | 1.00 |

Appendix 3

Physical properties of HMs and their compounds ^27^

| Metals | Melting Point, ℃ | Boiling Point, ℃ | Oxides, ℃ | Chlorides, ℃ | Sulfates, ℃ |
| --- | --- | --- | --- | --- | --- |
| Cr | 1900 | 2480 | m.p.2435, b.p.3000 | m.p.83 | m.p. 1350 |
| Ni | 1555 | 2837 | m.p. 1980 | m.p. 1001 | m.p. 99 |
| As | 817 | 613 | m.p.275_313, b.p.465 | m.p._18, b.p.130.2 | m.p.320, b.p.565 |
| Cd | 321 | 767 | m.p.900, b.p.1385 | m.p.570, b.p.960 | m.p.1000 |
| Ba | 725 | 1845 | m.p.1920, b.p.2000 | m.p.960, b.p.1560 | m.p.1200 |
| Hg | _39 | 357 | decomposable above 400 | m.p.275, b.p.301 | m.p.584 |
| Pb | 327 | 1744 | m.p.866, b.p.1516 | m.p.501, b.p.950 | m.p.1170 |
